# Malignant ascites: a source of therapeutic protein against ovarian cancer?

**DOI:** 10.18632/oncotarget.27185

**Published:** 2019-10-15

**Authors:** Pascale Ribaux, Aurore Britan, Gabriele Thumann, Florence Delie, Patrick Petignat, Marie Cohen

**Affiliations:** ^1^ Department of Pediatrics, Gynaecology and Obstetrics, Faculty of Medicine, Geneva 1206, Switzerland; ^2^ Translational Research Center in Oncohaematology, Faculty of Medicine, Geneva 1206, Switzerland; ^3^ Experimental Ophthalmology, University of Geneva, Geneva 1205, Switzerland; ^4^ Department of Ophthalmology, University Hospitals of Geneva, Geneva 1205, Switzerland; ^5^ School of Pharmaceutical Sciences, University of Geneva, University of Lausanne, Geneva 1205, Switzerland

**Keywords:** ascites, PEDF, sleeping beauty transposon, ovarian cancer, tumor development

## Abstract

Ovarian cancer is the fifth leading cause of cancer-related death in the world. Some ovarian cancer patients present large amount of ascites at the time of diagnosis which may play an active role in tumor development. In earlier studies, we demonstrated that the acellular fraction of ascites can induce apoptosis of ovarian cancer cells. The current study identifies pigment epithelium derived factor (PEDF) as the molecule responsible for the apoptotic effect of ascites and evaluates the Sleeping Beauty transposon (SBT) system as a new tool for PEDF gene therapy against ovarian cancer. We utilize gel filtration, mass spectrometry, affinity column, cell viability assay, tumor development on chick chorioallantoic membrane and molecular biology techniques for these purposes. PEDF was thus identified as the agent responsible for the effects of ascites on ovarian cancer cell viability and tumor growth. Interestingly, the PEDF expression is decreased in ovarian cancer cells compared to healthy ovarian cells. However, the level of PEDF is higher in ascites than in serum of ovarian cancer patients suggesting that cells present in the tumor environment are able to secrete PEDF. We then used the SBT system to stably induce PEDF expression in ovarian cancer cells. The overexpression of PEDF significantly reduced the tumor growth derived from these cells. In conclusion, the results presented here establish that PEDF is a therapeutic target and that PEDF from ascites or SBT could be utilized as a therapeutic strategy for the treatment of ovarian cancer.

## INTRODUCTION

Malignant ascites is a peritoneal exudate that results mainly from impaired drainage of the peritoneal cavity due to obstruction of lymphatic system by cancer cells and/or elevated increased filtration rate into the peritoneal cavity from the increased microvasculature lining the peritoneal cavity [1]. Ascites is composed of cellular and acellular fractions that are modulated during the course of the diseases in response to signals from the tumor and stromal cells [2]. Ascites is generally considered as contributing to cancer progression due to its growth factors, cytokines, chemokines and extracellular matrix components known to promote cell growth, invasion or resistance to apoptosis, and by its capacity to facilitate multifocal cancer cells dissemination [1]. However, malignant ascites also contains anti-angiogenic and proapoptotic factors that may reduce tumor development [1, 3].

Previously, we have reported that treatment with malignant ascites from high grade serous ovarian cancer patients induced apoptosis of ovarian cancer cells [4] and that this occurs through a JNK signaling pathway followed by increased levels of Fas, FasL and increased cytoplasmic localization of Daxx, suggesting the importance of the Fas/FasL pathway in ascites-induced apoptosis. Using ovarian tumor growth on the chick chorioallantoic membrane (CAM), we also demonstrated that ascites can decrease tumor growth significantly and increase response to paclitaxel (Ptx) treatment [4]. Together, these observations suggest that acellular fractions of ascites from high grade serous ovarian cancer patients may have a beneficial effect, particularly for patients treated with Ptx.

Here, malignant ascites were fractionated by gel filtration. The resulting fractions were tested for their capacity to alter cell viability. Then, inactive and active ascites fractions and unfractionated ascites were analysed to identify the potent proteins involved in apoptotic effects of malignant ascites. One of the protein found in the active fractions is pigment epithelium derived factor (PEDF), which is well-known to induce apoptosis *via* Fas/FasL pathway in endothelial cells (for review, see [5]) and is a potent inhibitor of angiogenesis [5, 6].

Since we have shown that PEDF expression is decreased in ovarian cancer cells compared to controls, SKOV3 cells were stably transfected with the *PEDF* gene using the SBT system to produce elevated PEDF levels and their ability to develop ovarian tumors was evaluated on the chick CAM model.

## RESULTS

### Candidate ascites proteins affecting SKOV3 cell viability

**Figure 1:**
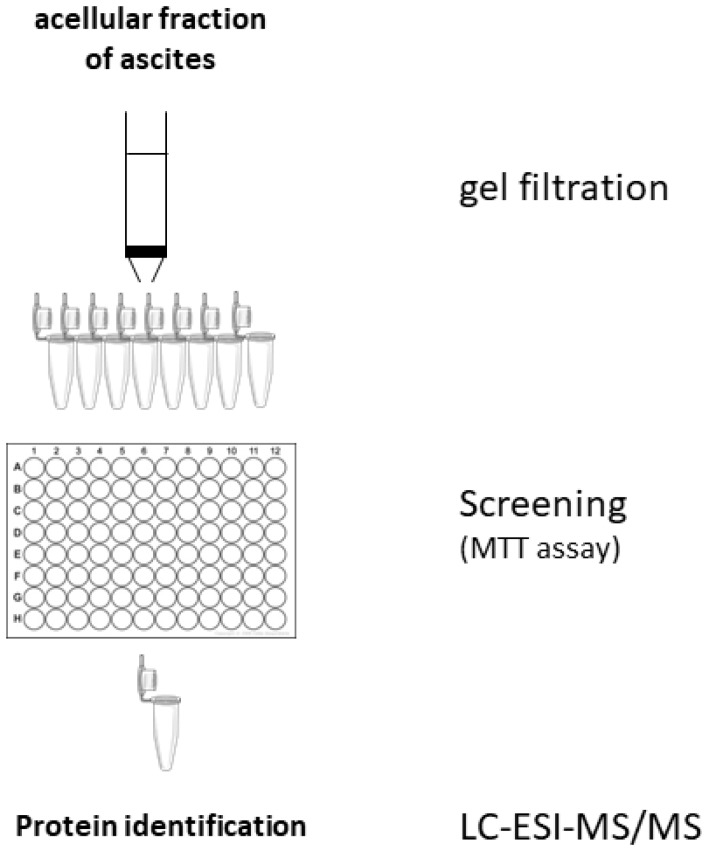
Scheme of the protocol used for identification of proteins responsible for the pro-apoptotic effects of ascites effects.

To identify candidate proteins responsible for the ascites effects, the experimental scheme of Figure 1 was followed. The ascites fluid was fractionated on a Sephadex G100 column. Each of the 10 fractions (F1 – F10) was evaluated for its effect on SKOV3 cell viability (Supplementary Figure 1). An analysis of the inactive fraction F2 and active fraction F10 by LC-ESI-MS/MS identified 4 proteins that are present in unfractionated ascites and active fraction F10 but not in inactive fraction F2 (Table 1). Of the four proteins, PEDF seems to be a good candidate for the suppressive effect of ascites on cell viability since PEDF is a known pro-apoptotic protein *via* Fas/FasL pathway activation [7–11].

**Table 1:** Proteins found in active and absent in inactive fractions of ascites

Proteins	Accession number	MW (kDa)	Linked to ovarian cancer	Linked to Fas/FasL apoptosis
Keratin, type I cytoskeletal 9	P35527	62		
Pigment epithelium-derived factor	P36955	46	[9] [11] [10]	[7] [8]
Protein AMBP	P02760	39		
Retinol-binding protein 4	P02753	23	[32] [33] [34]	

### PEDF level in control and cancer ovarian cells and fluids

**Figure 2:**
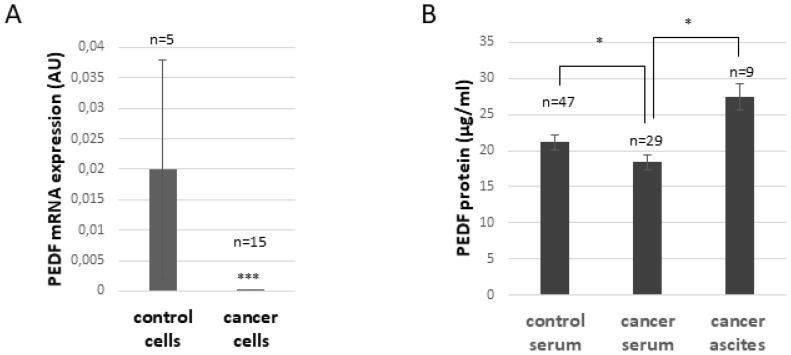
PEDF expression in normal and ovarian cancer patients. (**A**) PEDF mRNA expression in ovarian control or cancer cells. ^***^ *p* < 0.005 (**B**). PEDF protein level in serum of control, ovarian cancer patients, and in the acellular fraction of ascites obtained from ovarian cancer patients. ^*^ *p* < 0.05.

As illustrated in Figure 2A, PEDF mRNA expression is significantly decreased in ovarian cancer cells compared to control cells (*p* ≤ 0.005). PEDF protein level in serum is also slightly decreased in ovarian cancer patients compared to control patients (*p* ≤ 0.05, Figure 2B), whereas the level of PEDF in ascites is significantly higher compared to levels in serum from ovarian cancer patients (*p* ≤ 0.05; Figure 2B), suggesting that non-cancerous cells within the tumor environment secrete PEDF.

### Effect of recombinant PEDF (rPEDF) on ovarian cancer cell viability

**Figure 3:**
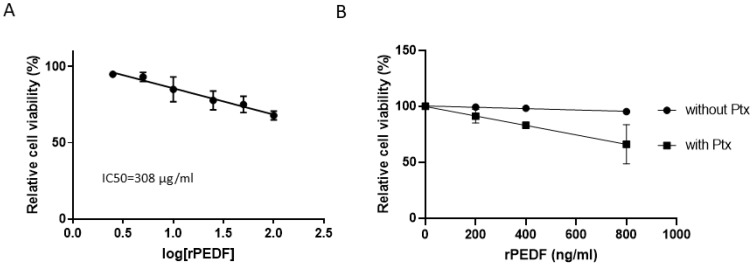
Dose-response effect of PEDF on SKOV3 cell viability in the presence and absence of Paclitaxel. (**A**) Dose-response effect of recombinant PEDF (rPEDF) on SKOV3 cell viability. The 50% inhibitory concentration (IC50) of recombinant PEDF was calculated using Compusyn software.(**B**) Determination of the minimal rPEDF concentration allowing additional effect with paclitaxel (Ptx at 100 nM, for 48h) on SKOV3 cell viability.

A dose-response analysis of the effect of rPEDF on SKOV3 cell viability showed an IC50 of 308 μg/ml (Figure 3A), a concentration that is much higher than those found in biological fluids. Figure 3B illustrates the synergism between PEDF and Ptx; at a concentration of 800 ng/ml rPEDF enhances Ptx effect on cell viability. Considering that ascites contain more than 25 μg/ml of PEDF (Figure 2B), the observation that 800 ng/ml PEDF is sufficient to significantly enhance the apoptotic effects of Ptx (*p* ≤ 0.05) substantiates the hypothesis that PEDF may be responsible for the apoptotic effects of ascites observed when the cells are treated with Ptx [4].

### Role of PEDF in the effects of ascites on ovarian cancer cell viability

**Figure 4:**
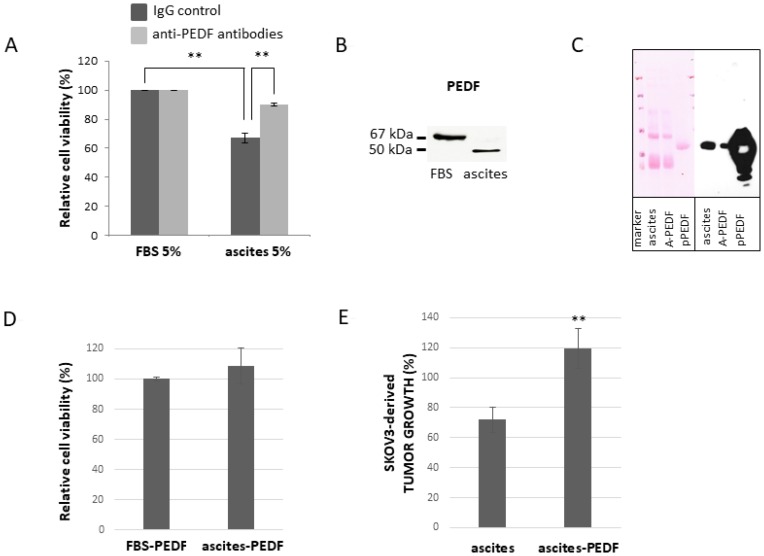
Effect of PEDF from malignant ascites on viability of SKOV3 cells. (**A**) Anti-PEDF antibodies (light grey bars) or control IgG (dark grey bars) were added in culture medium of cells treated with 100 nM Ptx. FBS: fetal bovine serum. (**B**) Western blot analysis of PEDF in FBS and ascites (**C**) Western blot analysis of PEDF in ascites, PEDF-depleted ascites (A-PEDF) and purified PEDF (pPEDF). Left panel: Ponceau red staining of membrane; Right panel: PEDF immunoblotting. (**D**) Effect of ascites-PEDF on viability of cells treated with 100 nM Ptx (for 48h) compared to FBS-PEDF. (**E**) Effect of PEDF depletion from ascites on Ptx treated SKOV3-derived tumor growth. ^**^ *p* < 0.01.

**Figure 5:**
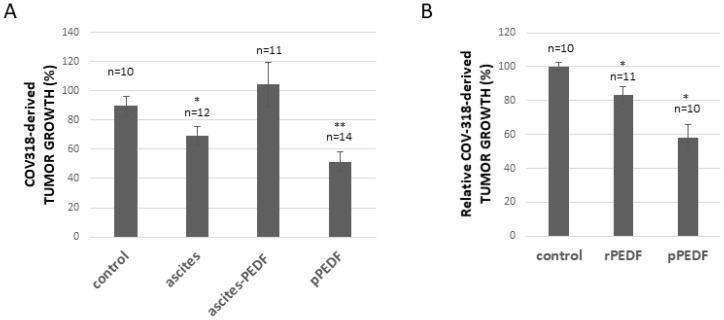
Effect of ascites and PEDF on COV318-derived tumor growth. (**A**) Evaluation of Ptx treatment in combination with ascites, PEDF-depleted ascites (ascites-PEDF) or purified PEDF (pPEDF) treatment on COV318-derived tumor growth. (**B**) Evaluation of recombinant PEDF (rPEDF) or purified PEDF (pPEDF) treatment on COV318-derived tumor growth. ^*^ *p* < 0.05 and ^**^ *p* < 0.01.

To evaluate the hypothesis that PEDF is responsible for the enhanced apoptotic effect of Ptx, we have treated SKOV3 cells with Ptx in the presence or absence of 5% ascites plus anti-PEDF antibodies (to neutralize PEDF secreted in the culture medium). As illustrated in Figure 4A, the addition of anti-PEDF antibodies does not affect cell viability when cells are cultured in 5% serum; however the addition of anti-PEDF to cells reduces the apoptotic effect of 5% ascites. We then analysed PEDF present in FBS and ascites by Western blot. As observed in Figure 4B, FBS and ascites contain PEDF, but PEDF isoform found in ascites seems to be different from the isoform observed in FBS. This may explain why ascites but not FBS can enhance the effect of Ptx on cell death (Figure 4A). To further substantiate the hypothesis that PEDF in ascites enhances the apoptotic effect of Ptx, ascites was partially depleted of PEDF (Figure 4C) and used to determine SKOV3 cell viability. As illustrated in Figure 4D, the addition of ascites partially depleted of PEDF, on SKOV3 cells treated with Ptx, has no further apoptotic effect compared to Ptx plus FBS treatment. Likewise, PEDF-depleted ascites does not enhance the Ptx inhibition of SKOV3-derived tumor growth on the CAM as does whole ascites (Figure 4E), which we have reported previously [4]. To confirm that the apoptotic enhancement effect of ascites is not specific to SKOV3-derived tumor growth, COV318-derived tumors grown on the CAM were treated with Ptx plus FBS (control), ascites, ascites-PEDF or purified PEDF (pPEDF). Figure 5A shows that (1) ascites induces a significant reduction of tumor growth, (2) depletion of PEDF from ascites abolishes the effect of ascites on tumor growth, and (3) pPEDF decreases the tumor size significantly. These results confirm the effects of ascites and PEDF on ovarian tumor growth. Since PEDF is a very potent anti-angiogenic factor, we investigated the effect of PEDF without Ptx treatment on tumor growth. As illustrated in Figure 5B, rPEDF decreased tumor size significantly. Purified PEDF from ascites decreased tumor size by 40%, demonstrating that PEDF exhibits antitumor activity independently of Ptx treatment *in ovo*.

### Therapeutic potential of PEDF gene therapy

Considering the effects of purified PEDF on ovarian tumor growth, it represents an attractive clinical tool against ovarian cancer and in fact PEDF has been proposed as a candidate anti-cancer agent against a range of tumors [5, 6].

**Figure 6:**
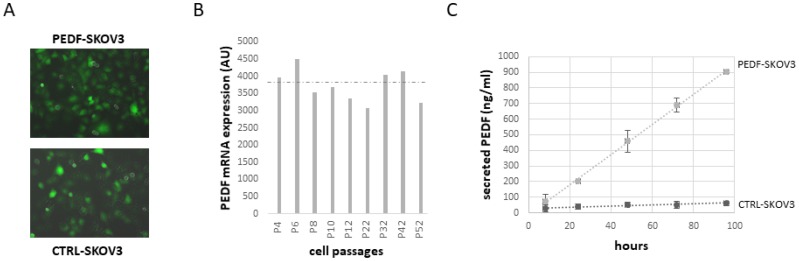
Expression of PEDF in SKOV3 cells stably transfected for PEDF with Sleeping Beauty Transposon (SBT) system. (**A**) GFP staining of SKOV3 cells stably transfected with PEDF or control transposon at passage 52 (P52). Magnification: ×200. (**B**) PEDF mRNA level in SKOV3 cells stably transfected with PEDF transposon at different passages (P). (**C**) Time-course of PEDF secretion in culture medium of SKOV3 cells stably transfected with PEDF transposon (grey) or control transposon (black) during 96 h of culture.

**Figure 7:**
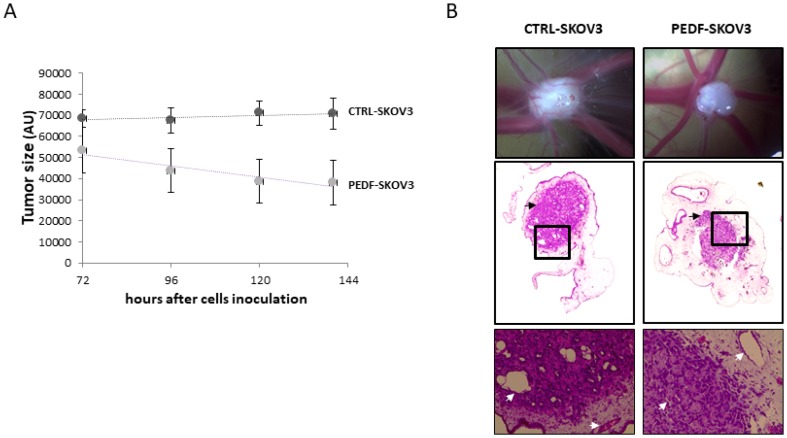
Tumorigenicity of PEDF-SKOV3 cells on CAM model. (**A**) Growth kinetic of PEDF-SKOV3 (grey) or CTRL-SKOV3-derived tumor (black). (**B**) Photographs (upper panel; magnification ×16.5) and Hemalun Eosin staining (middle panel; magnification ×100) of CTRL-SKOV3 and PEDF-SKOV3-derived tumors at EDD13.5. Enlargement of the black squares was presented in the lower panels (magnification ×400). Tumors are indicated by black arrows, and CAM and tumor vessels by white arrows.

However, no preclinical studies have been conducted to evaluate the therapeutic potential of PEDF against ovarian cancer. Since the half-life of PEDF is only about 4 h, it is not possible to deliver PEDF therapeutically over an extended period of time. One possibility would be to introduce the *PEDF* gene into the tumor cells for continuous expression. As proof of concept, SKOV3 cells have been transfected with a PEDF plasmid (PEDF-SKOV3) using the non-viral Sleeping Beauty transposon system and their ability to develop tumors has been evaluated. Control SKOV3 cells were transfected with the empty plasmid (CTRL-SKOV3). Figure 6A shows that after 6 months in culture, both PEDF-SKOV3 and CTRL-SKOV3 retain GFP staining, indicating stable transfection. The mRNA expression of PEDF in PEDF-SKOV3 cells is significantly increased compared to CTRL-SKOV3 cells that express a very low level of PEDF (too low to be observable on the graph, Figure 6B). Figure 6C illustrates the secretion of PEDF into the media by cultured PEDF-SKOV3 and CTRL-SKOV3 cells; note that PEDF-SKOV3 cells secrete significant amounts of PEDF, which accumulates reaching 900 ng/ml during 96 h of culture as the cells proliferate; control cells secrete very small amounts of PEDF and after 96 h in culture PEDF only reaches 60 ng/ml. We then evaluated the tumorigenicity of PEDF-SKOV3 cells on CAM model. As observed in Figure 7A, the initial size of PEDF-SKOV3-derived tumor is significantly lower than control cells and it decreased with time in contrast to CTRL-SKOV3-derived tumor size that slightly increased. Moreover, the vascularization of the tumor derived from PEDF-SKOV3 cells is less developed compared to tumor derived from CTRL-SKOV3 cells (Figure 7B).

## DISCUSSION

The data presented here confirm our previous results that ascites from ovarian cancer patients contain pro-apoptotic agents that can enhance the effect of Ptx *in vitro* and *in ovo* and that one of the pro-apoptotic agents is PEDF. This latter is a 50-kDa secreted glycoprotein belonging to the supergene family of serpins (serine protease inhibitors) that has potent anti-angiogenic, pro-apoptotic and neurotrophic activities [12, 13]. It is expressed by most normal and pathological vertebrate tissues and its effect is cell specific. To neurons, PEDF is neurotrophic, promoting cell survival and differentiation. However, when endothelial cells are exposed to PEDF, cell migration and proliferation are inhibited and the cells underdo apoptosis [13].

Due to its anti-angiogenic properties, several studies have demonstrated that PEDF can exhibit strong antitumor activity; however, the exact mechanism of PEDF action on tumor is not fully understood. The anti-angiogenic and neurotrophic activities seem to be linked to its N-terminal region. The deduced amino acid sequence contains consensus sequences for N-linked glycosylation and several predicted sites for phosphorylation (Ser-14, Ser-114 and Ser-227) and O-linked glycosylation [6]. The significance for some of these post-translational modifications is not fully elucidated but phosphorylation states of PEDF may differentially regulate its biological activity [14, 15]. Moreover, the different biological activities elicited by PEDF may be due to its binding to different PEDF receptors or proteins [6].

In ovarian tumor and ovarian cancer cells, PEDF expression is reduced or absent ([11], and Figure 2). Even though PEDF is reduced in tumors, it is relatively high in malignant ascites and higher than in serum, suggesting that the cells in tumoral microenvironment secrete PEDF.

Cheung and colleagues have shown that PEDF inhibited the proliferation and increased the apoptosis of SKOV3 cells [11]. Inhibition of endogenous PEDF with siRNA or neutralizing PEDF antibodies promoted the growth of these cells, indicating that the tumor-growth inhibition was a specific effect of PEDF. In the present study, the IC50 of rPEDF was approximatively 300 μg/ml or 6.2 mM, a much higher concentration than used in Cheung’s studies or found in biological fluids. Nevertheless, we found that rPEDF, in the concentration range of biological fluids, can enhance the effect of Ptx on cell viability. We also observed that the effect on tumor growth of rPEDF is reduced compared to the effect of pPEDF suggesting that post-translational modifications of PEDF protein (i.e. phosphorylation or glycosylation) or some cofactors eluted with PEDF from Sephadex column may enhance PEDF activity. Considering the importance of PEDF phosphorylation in anti-tumor activities [14, 15], we thus evaluated the serine phosphorylation of pPEDF from ascites, PEDF in ascites, and rPEDF (Supplementary Figure 2). We confirmed the absence of serine phosphorylation in rPEDF and its presence in PEDF isolated from ascites. However, we can neither confirm that these modifications are responsible for enhanced PEDF activity in our model nor exclude that other post-translational modifications or the presence of some cofactors eluted with PEDF from Sephadex column may be implicated.

Considering that PEDF is a potent anti-angiogenic factor that induces ovarian cancer cell apoptosis and that anti-angiogenesis is an effective therapeutic strategy for ovarian cancer treatment [16], PEDF represents an exceptional candidate anti-cancer agent. Three main strategies have been developed to increase PEDF level in the tumor environment:

1. systemic administration of PEDF protein or peptides [8, 17–21], however, the short half-life of PEDF makes administration of PEDF protein impractical

2. PEDF additive gene therapy [22–27] and

3. cell-based therapy using *PEDF* virally infected human mesenchymal cells [28–30].

Despite these studies confirmed the interest of PEDF therapy against cancer, delivering an effective concentration of PEDF locally within the tumor environment presents difficulties that must be solved before PEDF therapy can become clinically applicable. Moreover, due to the different roles of PEDF in physiological and pathological tissue, before its use as anti-cancer agent, it is imperative to evaluate its potential adverse effects on physiological tissues and/or to specifically deliver PEDF therapy in cancer cells.

We have shown here that integrating the PEDF transgene into SKOV3 ovarian cancer cells’ genome using the SBT system is feasible. The PEDF- SKOV3 cells expressed serine-phosphorylated form of PEDF (Supplementary Figure 2) and maintained PEDF expression for the 6 months that they were cultured. And more importantly when they were implanted on the CAM, they formed smaller tumors than controls and decreased in size with time. The decrease in tumor size observed from day 3 to day 6 may be the result of the SKOV3 cells having proliferated and reached a critical number to produce a level of PEDF effective at self-apoptosis.

## MATERIALS AND METHODS

### Ethical committee

The study was approved by Ethics Committee of Maternity and Pediatrics, University Hospital of Geneva (Geneva, Switzerland). Written informed consent was obtained from patients prior to enrollment in the present study.

### Ascites

Ascites, routinely obtained by paracentesis from ovarian cancer patients, was immediately centrifuged at 600 g for 8 min at room temperature. The supernatant, i.e. the acellular ascites fraction, was collected and kept at −20° C until use.

### Gel filtration

Ascites samples, 2-fold concentrated using Amicon 3 kDa (Merck Millipore, Germany), and diluted 1:1 (v:v) in sodium phosphate buffer 0.1 M, pH7.2, was applied to Sephadex G100 (GE healthcare, UK) column (Biorad) equilibrated in sodium phosphate buffer 0.1 M, pH7.2.

Ten fractions (F1 to F10) were collected (one every 10 min) and kept at −20° C until use.

### Liquid chromatography-electrospray ionization-mass spectrometry/MS (LC-ESI-MS/MS)

#### Digestion of proteins

10 μg total protein from each fraction were dissolved in 100 μl of 6 M urea + 50 mM ammonium bicarbonate and incubated at 37° C for 30 min followed by reduction with 2 μl of 50 mM dithioerythritol in distilled water for 1 hour at 37° C and alkylation with 2 μl of 400 mM iodoacetamide in distilled water) for 1 h at room temperature in the dark with agitation. The reduced and alkylated proteins were diluted 3-fold with 50 mM ammonium bicarbonate containing 1 ug trypsin porcine (solution sequence grade modified; Promega Corporation, Madison, WI, USA); after overnight digestion at 37° C, the samples were desalted using a C18 microspin column (Harvard Apparatus, Holliston, MA, USA), dried and dissolved in 5% CH_3_CN/0.1% formic acid prior to LC-ESI-MS/MS analysis.

#### Peptide fragmentation sequencing

LC-ESI-MS/MS was performed using a linear trap quadrupole (LTQ) Orbitrap Velos (Thermo Fisher Scientific, Inc.) equipped with a NanoAcquity system (Waters Corporation, Milford, USA). Peptides were trapped on a 5 μm 200 Å Magic C18 AQ (Bruker-Michrom, Auburn, CA, USA) 0.1 × 20 mm pre-column and were separated using a commercial 0.075 × 150 mm analytical nanocolumn (C18, 5 μm, 100 Å; Nikkyo Technos Co., Ltd., Japan). Peptides separation was performed by step gradient using H_2_O/formic acid 99.9%/0.1% (solvent A) and CH_3_CN/formic acid 99.9%/0.1% (solvent B). The gradient steps were 0-1 min 95% A and 5% B, followed by 55 min of 65% A and 35% B for 55 min followed by 65 min of 20% A and 80% B for 65 min, at a flow rate of 220 nl/min. ESI was performed at atmospheric pressure in a positive ionization mode, without nebulizing gas. For MS analysis, the orbitrap resolution was set at 60,000 and the ion population was set at 5 × 10^5^ with an m/z window of 400–2,000. For protein identification, ≤8 precursor ions were selected for collision-induced dissociation (CID) in the LTQ. The ion population was set at 1 × 10^4^ (isolation width of 2 m/z), whereas for MS/MS detection in the orbitrap, it was set at 1 × 10^5^ with an isolation width of 2 m/z units. The normalized collision energies were set to 35% for CID.

#### Protein identification

Peak lists were generated from raw orbitrap data using the EasyProtConv conversion tool (version 1.6) from the EasyProt software platform. The peak list files were searched against the SwissProt database (release 15.10 of 21-Sept-2011) using Mascot (version 2.2.0; Matrix Science, Ltd., London, UK). Human taxonomy (20,323 sequences) was specified for database searching. The parent ion tolerance was set to 10 ppm. Amino acid modifications were oxidized using methionine and carbamidomethyl cysteine. Trypsin was selected as the enzyme, with one potential missed cleavage, and the normal cleavage mode was used. The Mascot search was validated using Scaffold 3.6.5 (Proteome Software, Portland, OR, USA). Proteins matching two alternate peptides with a minimum probability score of 95% were selected for further analysis.

### Purification of PEDF from ascites

PEDF was purified from ascites using an anti-PEDF affinity column prepared using Aminolink plus Immobilization kit (ThermoFisher Scientific, Switzerland) and anti-PEDF antibody (LS-C100901, LSBio, Labforce, Switzerland) following the provider’s instructions. After equilibration of the anti-PEDF column with phosphate buffered saline (PBS) pH7.2, 1 ml of ascites, diluted 1:1 (v:v) with PBS (1:1, v:v) was loaded on the affinity column and allowed to equilibrate for 30 min at room temperature. The column was centrifuged and the the flow-through was considered as PEDF depleted ascites: “ascites-PEDF”); the column was washed with 1 ml of PBS and centrifuged again. The resin was then washed with 2 ml of PBS and centrifuged 4 more times. Purified PEDF (pPEDF) was eluted with 1 ml of Elution Buffer (0.2 M glycine, pH2.5) into a centrifuge tube containing 50 μL of Neutralization Buffer (1 M Tris HCl, pH8.5) and centrifuged. Ascites, “ascites-PEDF”, and pPEDF were kept at −20° C until use.

### Cell culture

#### Ovarian cancer cell lines

Human ovarian carcinoma cell lines derived from ascites COV318 (ECACC, Sigma-Aldrich) and SKOV3 (ATCC) were cultured respectively in DMEM or RPMI (Gibco, Life Technologies, Switzerland) medium supplemented with 10% Fetal Bovine Serum (FBS, Biochrom AG, Switzerland) and 0.05 mg/ml gentamicin (Gibco, Life Technologies, Switzerland) at 37° C in a humidified atmosphere of 95% air and 5% CO_2_.

#### Ovarian primary cells

Human ovarian high-grade serous carcinoma and healthy human ovarian cells were isolated as previously described [31]. Briefly, ovarian tissue was digested with 4 mg/ml dispase (Gibco, Life technologies, Switzerland) in HBSS-Hepes (filtered on 0.22 μm) containing 1 μg/ml DNase (Roche, Diagnostics GmbH, USA) for 30 minutes at 37° C. Ovarian tissue and supernatant were placed in a 10 cm dish and tissue was scrubbed with a scalpel. Then, the supernatant was collected, neutralized with 5% FBS, filtered through a 100 μm mesh (BD Biosciences, San Jose, USA) and centrifuged at 800 g for 8 min. The pellet was resuspended in DMEM-10%FBS-0.05 mg/ml gentamicin and seeded in 3 cm dish. Cells were characterized by PCR for CD90, HE4, PAX8, cytokeratin 8, cytokeratin 19, cytovillin and by immunocytochemistry for cytokeratin 7, cytokeratin 18, cytokeratin 19, vimentin, p53 (data not shown). Cells were used before passage 5.

### Cell viability

SKOV3 cell viability was evaluated using the methylthiazolyldiphenyl-tetrazolium bromide (MTT) assay.

Ovarian cancer cells were seeded in 96-well plates at a density of 30 000 cells/well. After 24 h, cells were treated with culture medium supplemented with either recombinant PEDF (rPEDF) (BioProductsMD, XpressBio Europe), ascites, ascites fraction (5% equivalent ascites) or “ascites-PEDF”, with or without 100 nM Ptx (Sigma-Aldrich, Switzerland) for 48 h. Culture medium was then replaced by 100 μl of MTT reagent (Sigma-Aldrich, Switzerland). After 2 h incubation at 37° C, 150 μl of MTT solvent was added and plates were agitated for 15 min at room temperature. Absorbance was measured at λ = 540 and 690 nm with a microplate reader. Cell viability was calculated by setting the data from normal culture medium as equivalent to 100% survival rate. The 50% inhibitory concentration (IC50) of recombinant PEDF was calculated using Compusyn software.

### Establishment of stable SKOV3 cells overexpressing PEDF

#### Plasmids

Plasmids used were pCMV3-SERPINF1 (# HG11104-UT, from Sino Biological Inc, USA), pSBbi-GN (# 60517, from Addgene, MA, USA (contains a GFP tag)) and pCMV(CAT)T7-SB100 (# 34879, from Addgene, USA). The plasmid pSBbi-GN was the control (CTRL) transposon and pCMV(CAT)T7-SB100 was the transposase construct. The plasmid construct pSBbi-GN-PEDF, the PEDF transposon, was generated by cloning the insert of the human PEDF sequence from pCMV3-SERPINF1 into the HindIII-XbaI restriction site of pSBbi-GN.

#### Stable transfection and selection of cells

SKOV3 cells were seeded at a density of 250 000/well in 6-well plates and co-transfected with transposase and either CTRL or PEDF transposon using JetPei (Polyplus transfection, France) as a reagent (2 μl/μg of DNA). The ratio transposase/transposon used was 1:16 with a total DNA amount of 3 ug.

CTRL transposon or PEDF transposon transfected cells (respectively CTRL-SKOV3 and PEDF-SKOV3 cells) were grown in RPMI medium supplemented with 10% FBS and 0.05 mg/ml gentamicin for 6 days. Antibiotics selection with G-418 (Roche, Sigma-Aldrich, Switzerland) was performed at 1 mg/ml for 12 days until all cells were GFP positive. The G-418 selection was thereafter maintained at 0.1 mg/ml. Analysis of the PEDF mRNA expression was performed during 6 months from passage 4 (P4) to passage 52 (P52) with cells passed twice a week.

### Immunoprecipitation

Immunoprecipitation was performed overnight under shaking at 4° C on 1 ml of ascites or 450 mg of PEDF-SKOV3 total proteins with either 2 mg of mouse anti-PEDF antibodies (D-10, Santa Cruz Biotechnology, Germany) or 5 mg of rabbit anti-Phosphoserine antibodies (SAB5200086, Sigma-Aldrich, Switzerland). Then, protein A+G agarose suspension (IP05, Calbiochem, Switzerland) was added at the rate of 10 ml/mg of antibodies for 2 h under shaking at 4° C. Immunoprecipitated proteins were washed 4 times in lysis buffer containing a cocktail of proteases inhibitors (Complete Protease Inhibitor Cocktail, Merck, Switzerland, + 1 mM Na_3_VO_4_). Finally, Sample Buffer 2X was added and the samples were boiled 5 min at 95° C, centrifuged 1 min at 14 000 rpm and the supernatant was loaded on a 10% acrylamide gel.

### Western blot analysis

#### For PEDF expression in biological samples

FBS, pPEDF, ascites and “ascites-PEDF” were loaded on a Pierce Top 2 abundant proteins depletion column (ThermoFisher Scientific, Switzerland) following the provider’s recommendation to remove albumin and immunoglobulins before Western blot analysis. Proteins (10 μl FBS or ascites equivalent) were fractionated by SDS-PAGE and transferred to nitrocellulose membrane for immunoblot analysis using rabbit anti-PEDF antibodies (H-125, 1:1000 dilution from Santa Cruz Biotechnology, Germany) as primary antibodies and goat anti-rabbit IgG (H+L)-HRP conjugate (170-6515, 1:3000 dilution from Bio-Rad, Basel, Switzerland) as secondary antibodies. Specific signal was detected using Amersham ECL Prime Western Blotting Detection Reagent (Ge Healthcare, UK).

#### For immunoprecipitation

Immunoprecipitated proteins, 500 ng rPEDF or 140 ng pPEDF were fractionated by SDS-PAGE on a 10% gel, transferred to nitrocellulose membrane, blocked in PBS-3%BSA and incubated with either anti-PEDF antibodies (D-10, 1:500 dilution, for immunoprecipitation with anti-Phosphoserine antibodies) or anti-Phosphoserine antibodies (1:100 dilution, for immunoprecipitation with anti-PEDF antibodies). Signal was revealed by incubation in Clean-Blot IP detection (dilution 1:400, #21230 from ThermoFisher Scientific, Switzerland) for 1 h at room temperature, followed by Amersham ECL Prime Western Blotting Detection Reagent (Ge Healthcare, UK).

### RT-qPCR

RNA was extracted using PureLink^®^ RNA Mini kit (Ambion; Thermo Fisher Scientific, Inc.), according to the manufacturer›s instructions. Reverse transcription was performed with 1 μg total RNA in a final volume of 20 μl, using the High Capacity cDNA Reverse Transcription kit (Applied Biosystems; Thermo Fisher Scientific, Inc.). The quantitative detection of the PCR product was performed using a KAPA SYBR^®^FAST Universal qPCR kit (KAPA Biosystems, Inc., Wilmington, MA, USA). The relative expression levels of PEDF mRNA were normalized to the housekeeping genes GAPDH, HPRT1 and Cyclophilin A. The primer sequences were as follows: PEDF-f 5′TTACGAAGGCAAGTCACCA3′; PEDF-r 5′TAAGGTGATAGTCCAGCGGG3′; GAPDH-f 5′CGACCACTTTGTCAAGCTCA3′; GAPDH-r 5′CCCT GTTGCTGTAGCCAAAT3′; HPRT1-f 5′ATGACCAGTC AACAGGGGAC3′; HPRT1-r 5′TGCCTGACCAAGGA AAGCAA3′; Cyclophilin A-f 5′TACGGGTCCTGGCATC TTGT3′ and Cyclophilin A-r 5′CCATTTGTGTTGGGT CCAGC3′.

### PEDF ELISA

PEDF protein was quantified using a human PEDF ELISA kit (BioProductsMD, XpressBio Europe) following the provider’s instructions.

### Tumor development on chick chorioallantoic membrane (CAM)

#### Eggs

Fertilized eggs (animal facility of the University of Geneva, Geneva, Switzerland) were incubated at 38° C at 80% relative humidity with periodic rotation. Rotation was stopped on egg development day (EDD) 4 and a hole was drilled at the narrow apex, which was sealed with adhesive tape On EDD8, the hole in the eggshell was enlarged to allow the access to the CAM. After gentle scratching of the CAM with a needle tip, 2 × 10^6^ COV318, SKOV3, CTRL-SKOV3 or PEDF-SKOV3 cells suspended in 30 ul Geltrex^®^ (Life technologies, Basel, Switzerland) were implanted on the CAM and the hole was sealed with Parafilm^®^. On EDD11 tumor growth was monitored using a Wild Heerbrugg M3Z microscope at 10× magnification with a Lumenera INFINITY2-1 CDD camera equipped with Infinity Capture Software.

#### Treatment

Three days after cell implantation (EDD11), tumors were treated topically with pPEDF (1.25 μg/ml, corresponding to PEDF concentration in 5% ascites), ascites (5%), “ascites-PEDF” (5% equivalent ascites) or FBS (5%) with 100 μM Ptx. CAMs with untreated implanted cells served as controls. On EDD13 tumor size was monitored using a Wild Heerbrugg M3Z microscope at 10× magnification with a Lumenera INFINITY2-1 CDD camera equipped with Infinity Capture Software.

#### Histology

On EDD13 tumors were harvested, fixed in histofix for 24 h and paraffin-embedded for further histological and immunohistochemical analysis. Cell morphology was assessed on hematoxylin and eosin stained 8 μm sections.

### Statistical analysis

Results are expressed as mean ± SE. The differences between samples were evaluated by Student’s *t* test (unless otherwise specified) and *p* value ≤ 0.05 was considered significant.

## SUPPLEMENTARY MATERIALS





## Abbreviations

CAM: chorioallantoic membrane
EDD: egg development day
FBS: fetal bovine serum
LC-ESI-MS/MS: liquid chromatography electrospray ionization mass spectrometry
PBS: phosphate buffered saline
PEDF: pigment epithelium derived factor
pPEDF: purified PEDF
rPEDF: recombinant PEDF
Ptx: paclitaxel
SBT: Sleeping Beauty transposon
