# Supplementary material for: Malignant ascites: a source of therapeutic protein against ovarian cancer?
Source: Oncotarget. 2019 Oct 15;10(57):5894–905. doi: 10.18632/oncotarget.27185 (PMC6800269; doi:10.18632/oncotarget.27185)
Supplement: Supplementary file 1 [file oncotarget-10-5894-s001.pdf]

Malignant ascites: a source of therapeutic protein against ovarian cancer?

SUPPLEMENTARY MATERIALS

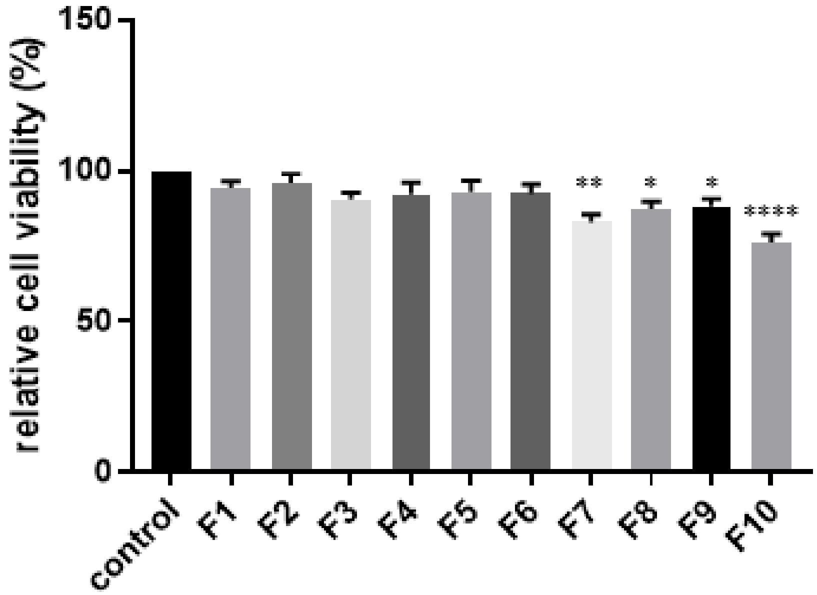

Supplementary Figure 1: Evaluation of ascites' fractions (F1 to F10) on SKOV3 cell viability. F1 to F6: inactive fractions; F7 to F10: active fractions compared to FBS (= control). $^{*}p < 0.05$, $^{**}p < 0.005$, $^{****}p < 0.00001$ (one way ANOVA test).

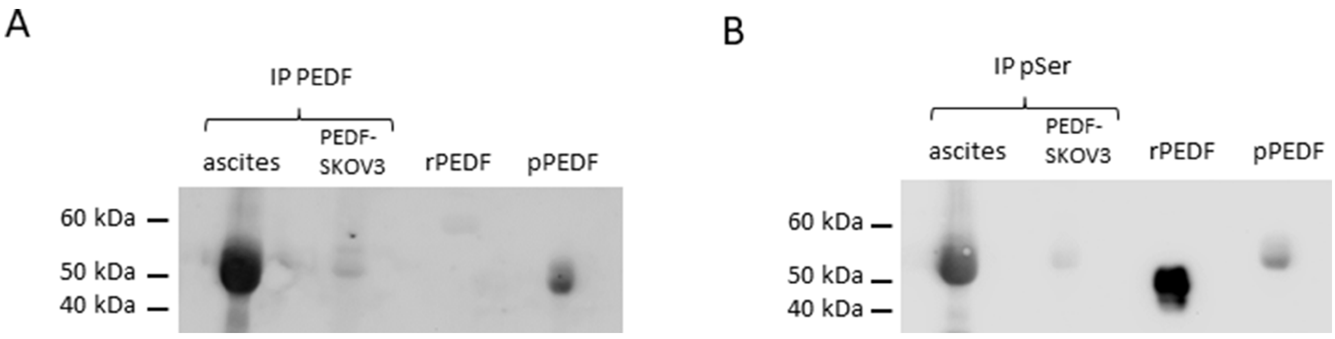

Supplementary Figure 2: Phosphorylation of serine residue of PEDF. Ascites or PEDF-SKOV3 total proteins were immunoprecipitated with either anti-PEDF antibodies (**A**) or anti-Phosphoserine antibodies (**B**). Immunoprecipitation products, rPEDF and pPEDF were immunoblotted with either anti-Phosphoserine antibodies (A) or anti-PEDF antibodies (B).

Supplementary Table 1: Proteins identification in ascites (A100), inactive fraction (F2) and active fraction (F10) In grey: proteins found in all samples; in dark grey: proteins found in ascites and F10. See Supplementary Table 1
